# Editorial: Emerging New Tests and Their Impact Upon the Practice of Reproductive Genetics

**DOI:** 10.3389/fgene.2021.828202

**Published:** 2021-12-17

**Authors:** Kwok-yin Leung, Antoni Borrell, Mark I. Evans, Ming Chen

**Affiliations:** ^1^ Department of Obstetrics and Gynecology, University of Hong Kong, Hong Kong, China; ^2^ Maternal Fetal Medicine Center, Gleneagles Hospital Hong Kong, Hong Kong, China; ^3^ BCNatal, Hospital Clínic Barcelona, Universitat de Barcelona, Barcelona, Spain; ^4^ Comprehensive Genetics, New York, NY, United States; ^5^ Department of Obstetrics and Gynecology, Icahn School of Medicine at Mt. Sinai, New York, NY, United States; ^6^ Department of Genomic Medicine, Changhua Christian Hospital Medical Center, Changhua, Taiwan; ^7^ College of Medicine, National Chung Hsing University, Taichung, Taiwan; ^8^ Department of Obstetrics and Gynecology, College of Medicine, and Hospital, National Taiwan University, Taipei, Taiwan; ^9^ Department of Medical Science, National Tsing Hua University, Hsinchu, Taiwan; ^10^ Department of Biomedical Science, Dayeh University, Changhua, Taiwan

**Keywords:** reproductive genetics, aneuploidy screening, expanded carrier screening, chromosomal microarray analysis, whole-exome sequencing, balanced chromosomal abnormalities, early pregnancy loss, preimplantation genetic testing

There has been a geometric explosion in genetic capabilities such that currently a wide variety of prenatal screening and diagnostic testing for fetal chromosomal abnormalities are available. Ideally, all woman should be counseled in each pregnancy about the benefits and limitations of available testing (American College of Obstetricians and Gynecologists’ Committee on Practice Bulletins—Obstetrics, 2020). In countries where equity and budgets are prioritized over personal choice by the various National Health Services, testing is offered only when a specific risk cut-off is reached. In the present issue, Antoni Borrell enlightens us on the impact of health education on informed decision. A randomized controlled trial was conducted on 160 pregnant women undergoing first trimester aneuploidy screening (Miño et al.). After receiving an extensive counseling, more women opted for an invasive prenatal testing while less women opted for the first trimester combined test and cell free DNA testing, as compared to those without extensive counseling (Miño et al.).

Recently, American College of Medical Genetics and Genomics (ACMG) recommends a consistent and equitable approach for offering carrier screening to all women during pregnancy or before conception (Gregg et al., 2021). Next-generation sequencing (NGS) allows identification of sequence variants across many genes and hence screening for multiple genetic conditions at the same time. A questionnaire survey involving 623 Chinese pregnant women and 300 nonpregnant women to study views and acceptance of expanded carrier screening (ECS) was reported in the present issue (Cheng et al.). Although more non-pregnant women accepted ECS compared to pregnant women (70.7 vs. 61.2%), fewer non-pregnant women heard about ECS than pregnant women (32.3 vs. 42.4%). The majority of them showed a lack of understanding about ECS despite being given pamphlets (Cheng et al.). It is important to improve women’s understanding of reproductive risk before making an informed decision, especially the issue related to false-negatives, of which the readers can refer to a recent excellent review (Evans et al., 2021).

While prenatal chromosomal microarray analysis (CMA) is a current standard genetic testing, prenatal whole exome sequencing and eventually whole genome sequencing are emerging technologies that will become front line tests in the future. CMA can detect major chromosomal imbalances as well as copy number variations that are too small to be detected by traditional karyotyping. Diagnostic sequencing is useful for evaluation of fetuses for whom CMA is uninformative. It may be offered concurrently according to accepted practice guidelines. CMA is less optimal than sequencing for the presenting fetal phenotype according to expert genetic opinion (International Society for International Society for Prenatal Diagnosis, 2018). Interpretation of variants found by sequencing is challenging because there is currently limited genotype–phenotype correlation in the prenatal setting. A case report in the present issue illustrates challenges of prenatal counseling due to the complexity of genomic data.

The prenatal identification of a baby with a MECP2 missense mutation and 15q11.2 microduplication in a family with a child having developmental epileptic encephalopathy associated with a *de novo* KCNQ2 mutation was reported (Huang et al.). The authors used an extended segregation analysis including the parents and their relatives to provide further useful information for genetic counseling.

Another limitation of prenatal sequencing is long turn-around time. In the present issue, prenatal diagnosis of two families with recurrent oligohydramnios by rapid trio-whole-exome sequencing (WES) which revealed mutations in the AGT gene within 1 week was reported (Lin et al.). A compound heterozygous mutation with c.856 + 1G > T and c.857-619_1269 + 243delinsTTGCCTTGC changes were found in the first family while homozygous c.857-619_1269 + 243delinsTTGCCTTGC mutations were found in the second family. These mutations are associated with autosomal recessive renal tubular dysgenesis which is a severe disorder with an unfavorable outcome. Notably, a few earlier reports from Taiwan had pointed out that the mutant allele c.857-619_1269 + 243delinsTTGCCTTGC of the AGT gene is very likely to be a founder variant in the Chinese population, which may aid in the prompter identification of the molecular pathology in that rapid trio-WEs case (Ma et al., 2019; Tseng et al., 2020). Such founder effect alleles are not uncommon in the Chinese population and had been reported previously at other inherited disorders such as the c.509G > A/p.G170D mutation of the PPIB gene causing the osteogenesis imperfecta IX in the Chinese population, and the way to estimate the mutation dating was also reported (Chang et al., 2020; Zhu et al.).

Recently, whole-genome sequencing (WGS) has been used to delineate the breakpoints of balanced chromosomal abnormalities (BCA) which have no visible gain or loss of genetic material at cytogenetic resolution (Redin et al., 2017). The use of whole-genome sequencing can detect complexity of BCA that is cryptic to karyotyping, and hence improve prediction of clinical outcomes for balanced rearrangements (Redin et al., 2017). In the present issue, a pilot project was reported, using short read genome sequencing (GS) to retrospectively re-sequence ten prenatal subjects with *de novo* BCAs (Yu et al.). GS enabled accurate identification of all breakpoints in these ten cases, revised the conventional karyotype results in nine cases, and provided additional information and changed the interpretation of the BCAs in four cases (Yu et al.). These results show us the importance of precisely delineation of breakpoints as BCAs are associated with neurodevelopmental disorders including intellectual disability and autism spectrum disorder.

Chromosome analysis of both couples with recurrent pregnancy loss (RPL) and products of conception (POC) is most informative in the investigation of possible genetic causes of RPL (Papas and Kutteh, 2021). Parental karyotyping can detect translocation carriers in 2–5% of RPL couples (Papas and Kutteh, 2021), while POC can demonstrate chromosomal anomalies in half of the cases. In the present issue, a new stepwise molecular work-up to diagnose chromosomal abnormalities in women who have had one or more early pregnancy losses was reported (Pauta et al.). This new molecular work-up included two quantitative fluorescence PCR (QF-PCR) rounds, and a high-resolution SNP-array in those cases with normal QF-PCR results. QF-PCR and CMA can overcome the pitfalls of conventional karyotyping including culture failure and submicroscopic abnormalities. Besides, the authors also proposed transcervical chorionic villus sampling (CVS) during surgical or before medical uterine evacuation to avoid maternal contamination. They found that transcervical CVS was more effective in the retrieval of embryonic tissue for chromosome analysis than examining POC after evacuation (Pauta et al.).

NGS after trophectoderm (TE) biopsy have been used for preimplantation genetic testing for aneuploidies (PGT-A) because of its high sensitivity (Popovic et al., 2020). Compared to CMA, NGS may detect more chromosome mosaicism in blastocysts which remains a perpetual diagnostic and clinical dilemma (Popovic et al., 2020). In the present issue, a retrospective cohort study on PGT-A from 4,036 blastocysts with NGS was reported (Chuang et al.). The authors explored whether the incidence of mosaicism for individual chromosome in blastocysts is correlated with chromosome length, highlighting the complex mechanisms of causing mosaicism in blastocysts.
